# Coevolving residues inform protein dynamics profiles and disease susceptibility of nSNVs

**DOI:** 10.1371/journal.pcbi.1006626

**Published:** 2018-11-29

**Authors:** Brandon M. Butler, I. Can Kazan, Avishek Kumar, S. Banu Ozkan

**Affiliations:** 1 Department of Physics and Center for Biological Physics, Arizona State University, Tempe, AZ, United States of America; 2 Harris School of Public Policy and Center for Data Science and Public Policy, University of Chicago, Chicago, IL, United States of America; Iowa State University, UNITED STATES

## Abstract

The conformational dynamics of proteins is rarely used in methodologies used to predict the impact of genetic mutations due to the paucity of three-dimensional protein structures as compared to the vast number of available sequences. Until now a three-dimensional (3D) structure has been required to predict the conformational dynamics of a protein. We introduce an approach that estimates the conformational dynamics of a protein, without relying on structural information. This *de novo* approach utilizes coevolving residues identified from a multiple sequence alignment (MSA) using Potts models. These coevolving residues are used as contacts in a Gaussian network model (GNM) to obtain protein dynamics. B-factors calculated using sequence-based GNM (Seq-GNM) are in agreement with crystallographic B-factors as well as theoretical B-factors from the original GNM that utilizes the 3D structure. Moreover, we demonstrate the ability of the calculated B-factors from the Seq-GNM approach to discriminate genomic variants according to their phenotypes for a wide range of proteins. These results suggest that protein dynamics can be approximated based on sequence information alone, making it possible to assess the phenotypes of nSNVs in cases where a 3D structure is unknown. We hope this work will promote the use of dynamics information in genetic disease prediction at scale by circumventing the need for 3D structures.

## Author summary

Proteins are dynamic machines that undergo atomic fluctuations, side chain rotations, and collective domain movements that are required for biological function. There is, therefore, a need for quantitative metrics that capture the dynamic fluctuations per position to understand the critical role of protein dynamics in shaping biological functions. A limiting factor in incorporating structural dynamics information in the classification of non-synonymous single nucleotide variants (nSNVs) is the limited number of known 3D structures compared to the vast number of available sequences. We have developed a new sequence-based GNM method, termed Seq-GNM, which uses co-evolving amino acid positions based on the multiple sequence alignment of a given query sequence to estimate the thermal motions of C-alpha atoms. In this paper, we have demonstrated that the predicted thermal motions using Seq-GNM are in reasonable agreement with experimental B-factors as well as B-factors computed using 3D crystal structures. We also provide evidence that B-factors predicted by Seq-GNM are capable of distinguishing between disease-associated and neutral nSNVs.

## Introduction

A 3D structure is still required to computationally obtain protein dynamics, drastically limiting the extent to which conformational dynamics can be incorporated into genomic analysis. The reason for this is that there are exponentially more sequences than experimental structures. Currently, UniProtKB contains more than 100 million sequence entries, whereas the PDB reports the number of known 3D structures to be around 140,000 [1]. Furthermore, the number of known sequences is increasing at an exponential rate, compared to the much slower addition of new experimental PDB structures. This is due to the advent of high-throughput genomic sequencing, which is providing an unprecedented amount of data for genomic analysis. The vast amount of sequence data has driven the rapid classification of novel genetic variations through genome-wide association studies [2,3]. A large catalogue of non-synonymous single nucleotide variants (nSNVs) occurs in coding regions that can severely impact protein function, potentially leading to disease [4]. There are many *in silico* methods developed using evolutionary methodologies such as positional conservation and phylogeny and those that combine evolutionary approaches with biochemical and structural properties to diagnose neutral and disease associated nSNVs [5–11]. However, the accuracy of the majority of these *in silico* prediction methods is significantly lower for predicting the impact of nSNVs at highly evolving sites [12–16]. Protein dynamics can also be used to elucidate the functional impact of nSNVs and mechanisms of disease [5,17]. Our previous studies have evinced that a site-specific conformational dynamics analysis is capable of diagnosing nSNVs irrespective of evolutionary conservation [5,18,19] and recently has been incorporated as an additional feature for *in silico* prediction tools [20]. However, only a small fraction of the catalogued nSNVs in the coding regions (i.e. missense variants) have 3D experimental structures, [20], impeding broad application of protein dynamics in *in silico* tool predictions.

Coevolution, on the other hand, has become a valuable tool for its ability to predict structural contacts of 3D structures, particularly using global information through Potts models [21–27]. Coevolving residues are inferred from a multiple sequence alignment (MSA) of a given protein family, whereby if two given amino acids exhibit concordant patterns of evolution throughout the MSA then they are assumed to be in close spatial proximity in the folded 3D structure. This evolutionary principle can be leveraged so that sequence information can be used to describe protein topology, making *de novo* structure prediction possible [24,27]. It has been reported that only one correct contact for every 12 residues in a protein is necessary for accurate topology-level modeling [28]. In addition to structure prediction, coevolution analysis has also been used to identify critical interactions between protein complexes [22] important functional sites [24] and allosteric response [29]. The use of coevolution for structure prediction is largely possible for two reasons. First, the amount of sequence data for different protein families is sufficient to be leveraged by this technique to make predictions. Second, the methods for inferring coevolving residues from an MSA are becoming increasingly robust [30–34].

Inferring evolutionary couplings from an MSA are based on two primary approaches categorized as local [35–37] and global approaches [37–39]. The global approaches detangle direct evolutionary couplings from indirect couplings which enables them to capture spatial contacts [40]. Regardless of the method, the accuracy of detecting coevolving residues that correspond to structural contacts is fundamentally limited by the number of sequence homologs in the MSA. While most of the current methods use only the sequence homologs of the protein family belonging to target sequence, integrating multiple orthology protein families (i.e. families that share similar phylogeny and retain similar functions) was used to increase the number of homologs to produce a more accurate statistical inference [41]. RaptorX, leverages this joint family methodology; it uses an ultra-deep neural network combining coevolution information with sequence conservation information to infer 3D contacts and has produced higher accuracy than other methods [42–44].

In this paper, we will demonstrate the efficacy of our novel sequence-based GNM approach, called Seq-GNM, to estimate the dynamics profile of a protein with no *a priori* knowledge of its 3D structure. This *de novo* approach based on a Gaussian network model (GNM) enables the prediction of the magnitude of mean-square fluctuations of residues, which are proportional to the B-factors determined by X-ray crystallography experiments. However, instead of using a cutoff distance to determine 3D contacts as does the original structure-based GNM, we use coevolving residues (evolutionary couplings) in our model. We show that the theoretical predictions from our Seq-GNM are in reasonable agreement with experimental crystallographic B-factors as well as the values obtained from the structure GNM models that use spatial contacts. We also extend this analysis to determine the capacity of our model to assess the functional impact of nSNVs. We will demonstrate that the dynamics predicted by Seq-GNM can adequately classify disease and benign nSNVs across the proteome.

## Results

### B-factor correlations: Sequence, structure, and experimental

**Fig 1:**
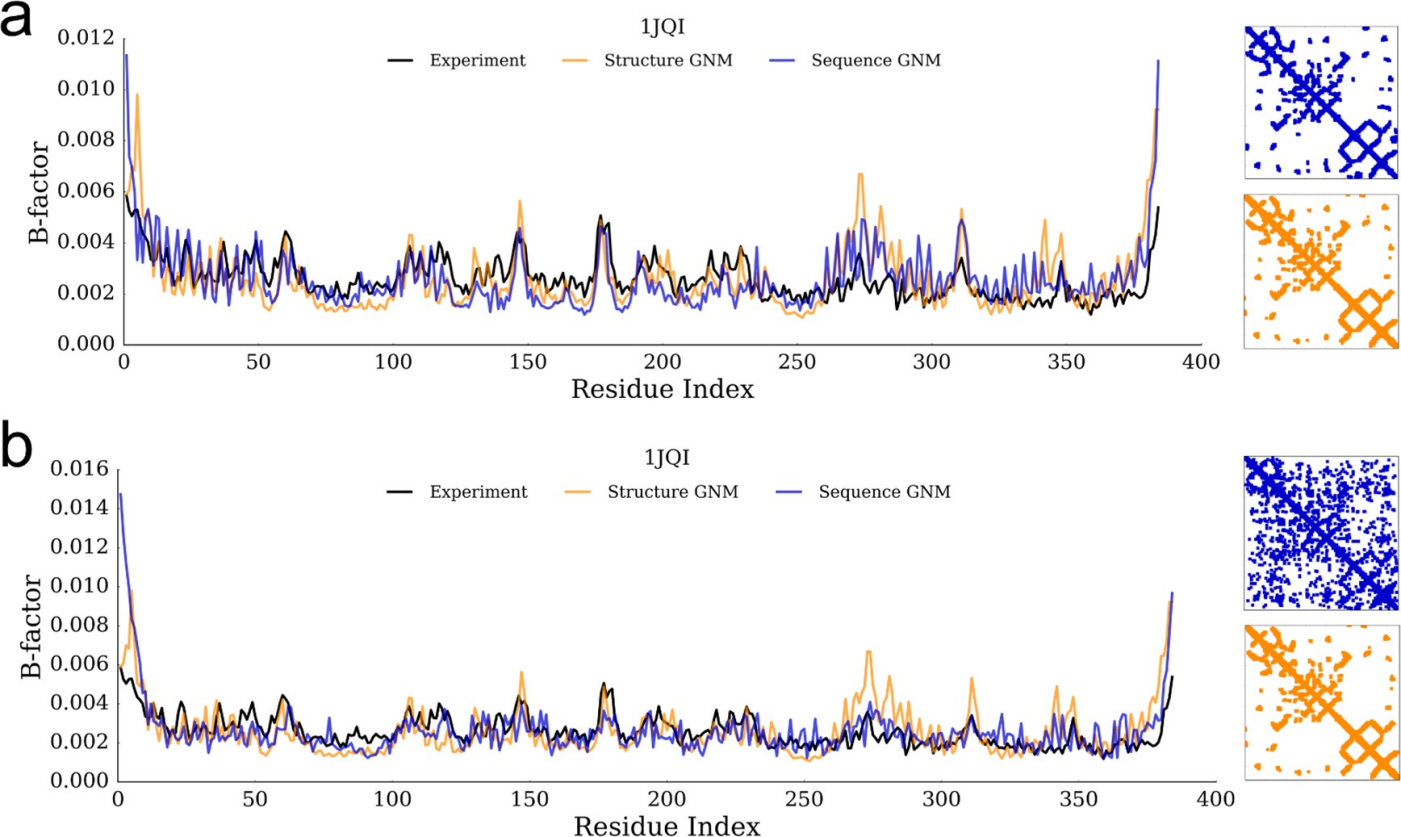
B-factors plots. A plot of theoretical B-factors as calculated by Seq-GNM (blue), the original GNM obtained from structure (orange), and observed experimental B-factors (black) for acyl-CoA dehydrogenase (PDB id: 1JQI) along with predicted contact maps by Seq-GNM (using a threshold score, shown as blue) and the contact map of the structure (using 10Å cut-off distance) (a) The Seq-GNM with values obtained from RaptorX produces a correlation of 0.56 with experiment, and 0.77 with the GNM obtained from structure. Moreover, the contact maps reveal that the predicted contacts between the Seq-GNM and structural GNM approaches are remarkably similar. (b) The Seq-GNM that uses values obtained from EVcouplings produces a correlation of 0.60 with experiment, and 0.68 with the GNM obtained from structure. The B-factor obtained by applying GNM to the experimental structure yields a correlation of 0.57. The contact map captures the dominant contacts with noise coming from poorly predicted EVcouplings scores.

We considered a high-resolution protein (2.25 Å) that is involved in amino acid catabolism, acyl-CoA dehydrogenase (1JQI), as an example case to examine the B-factor profiles and predicted contact maps using Seq-GNM. Coevolution analysis using direct coupling analysis (DCA) has been shown to recapitulate accurate structural contact maps for a wide range of proteins [21,23,24,27,31,45]. As expected, the contact maps of Seq-GNM and structural GNM are similar (Fig 1). In a comparison of their B-factor profiles, both Seq-GNM and structural GNM exhibit good agreement with observed B-factors, capturing flexible and rigid positions. Using evolutionary coupling (EC) values obtained from RaptorX, the correlation between the Seq-GNM and observed B-factors is 0.77, whereas the correlation between the structural GNM and observed B-factors is 0.57 (Fig 1A). Similarly, using EC values obtained by EVcouplings produced a correlation of 0.60 between the Seq-GNM and observed B-factors (Fig 1B). The scores obtained from EVcouplings are still reasonable, yet relatively lower correlations compared to those obtained by the RaptorX. This is likely due to the relatively noisy contact map predictions by EVcouplings compared to the more reliable contact maps produced by RaptorX (we think this is due to their inclusion of multiple orthology protein families) [42].

The Seq-GNM produces a correlation with crystallographic B-factors of 0.60, which is within the same range as those produced by the GNM from structure of 0.57. Moreover, theoretical B-factor profiles obtained from both methods were able to identify the catalytic sites on all of the proteins.

**Fig 2:**
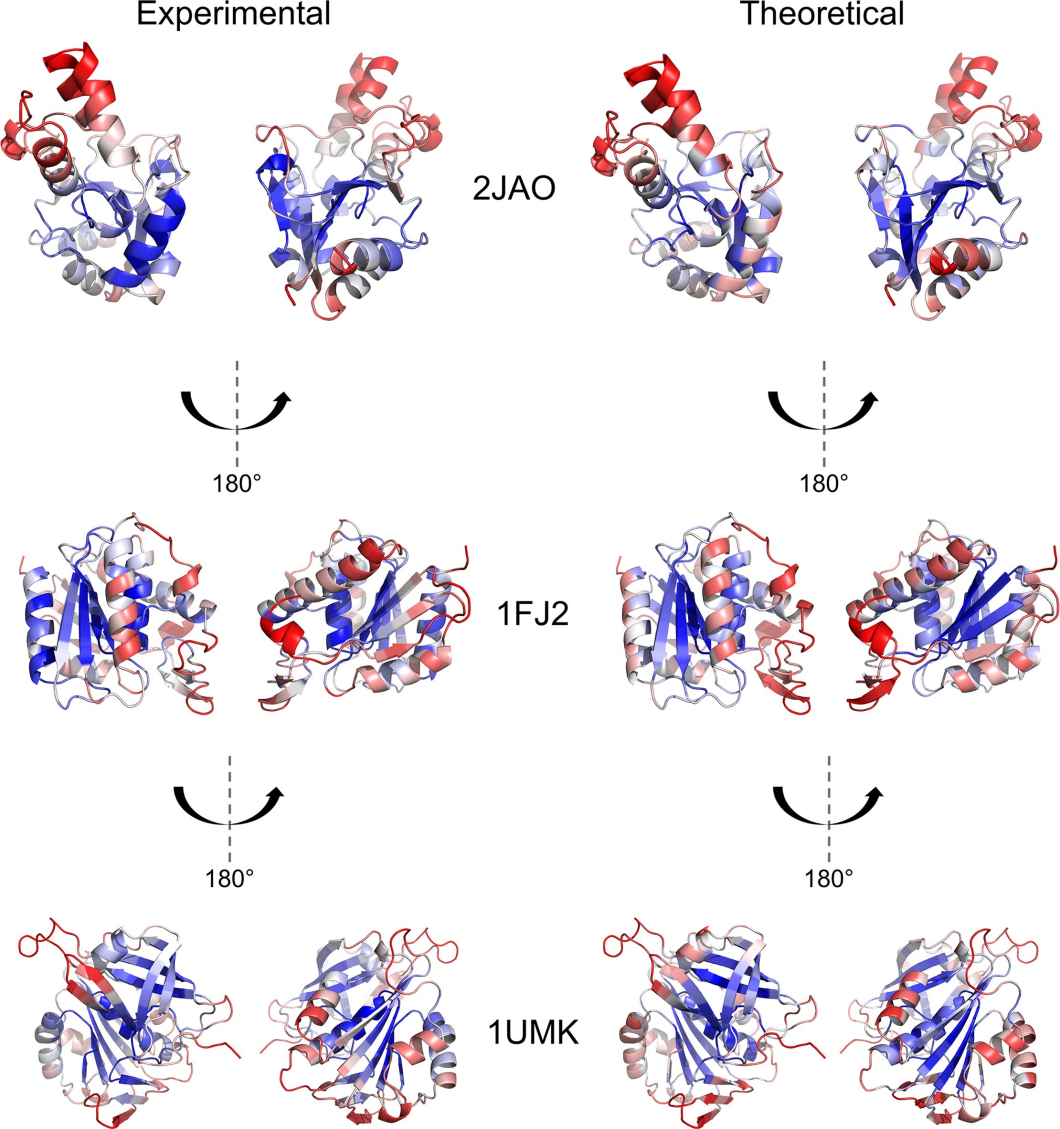
Color-coded ribbon diagrams using experimental and theoretical B-factors obtained by Seq-GNM. The observed crystallographic B-factors (left) and the predicted B-factors from the Seq-GNM superimposed on the structure. The three proteins selected–2JAO, 1F2J, and 1UMK–are high-resolution structures better than 2.0 Å. The B-factors are color-coded according to their B-factor profile on a spectrum of blue–white–red where blue represents the lowest B-factors (less mobility) and red represents the highest B-factors (more mobility). The B-factor scores are converted to a percentile rank so that they can be compared across different proteins. Each protein is also rotated 180° so that both sides can be visualized and compared. Moreover, the proteins are selected so that they have a variety of secondary structure components–2JAO contains primarily alpha helices, 1UMK is mainly composed of beta-sheets, and 1F2J is a combination of alpha helices and beta-sheets.

As a further test of the efficacy of the Seq-GNM, we superimposed the predicted B-factors onto the structures of three diverse proteins– 5'(3')-deoxyribonucleotidase (2JAO), acyl protein thioesterase (1FJ2), and NADH-cytochrome b(5) reductase (1UMK)–to visually contrast the predicted B-factors with that of experiment. Fig 2 shows each protein color-coded according to their B-factor profile on a spectrum of blue–white–red, where blue represents the lowest B-factors (less mobility) and red represents the highest B-factors (more mobility). The left panel shows the experimental B-factors for each protein, while the right panel shows the theoretical values predicted by the Seq-GNM. We investigated whether secondary structure was a factor in how the B-factors were distributed across the protein, and if certain secondary structure domains would exhibit less agreement with experiment. In this context, the proteins were selected so that they had a variety of secondary structure components–2JAO contains primarily alpha helices, 1UMK is mainly composed of beta-sheets, and 1F2J is a combination of alpha helices and beta-sheets. For 2JAO, the exterior helices that are flexible (red) in the observed structure are all reproduced in the predicted structure. The one highly rigid (blue) helix in the observed structure was more flexible in the predicted structure but was still in overall agreement. There is a surprising amount of similarity between the observed and predicted structure of 1F2J, considering that it contains both alpha-helix and beta-sheet elements. Similarly, 1UMK showed good agreement, except for some miniscule differences. This gives further evidence that the magnitudes of residue fluctuations predicted by the Seq-GNM model is representative of the crystallographic B-factor profiles for many proteins.

In order to compare predicted B-factors with crystallographic B-factors, we extracted a subset of 39 structures that had a resolution better than 2.0Å to obtain more realistic crystallographic B-factors (unreliable B-factors are common for many PDB structures) [18,46]. The same cutoff of 2.0Å was used in an earlier study to compare GNM predicted B-factors with those determined by crystallography [47]. For all 39 structures, the Seq-GNM (using EC values from RaptorX) and structure GNM were used to estimate their B-factors, which were then compared with the observed B-factors by calculating the correlation for each protein. The mean correlation coefficient for the Seq-GNM was 0.53 while the mean correlation coefficient for the structure GNM was 0.58. The correlation of 0.58 for structural GNM of our smaller data set is consistent with the findings of Kundu et al. where 113 high-resolution structures (resolution <2.0 Å) were used and, the mean correlation coefficient with observed B-factors was 0.59 [47].

**Fig 3:**
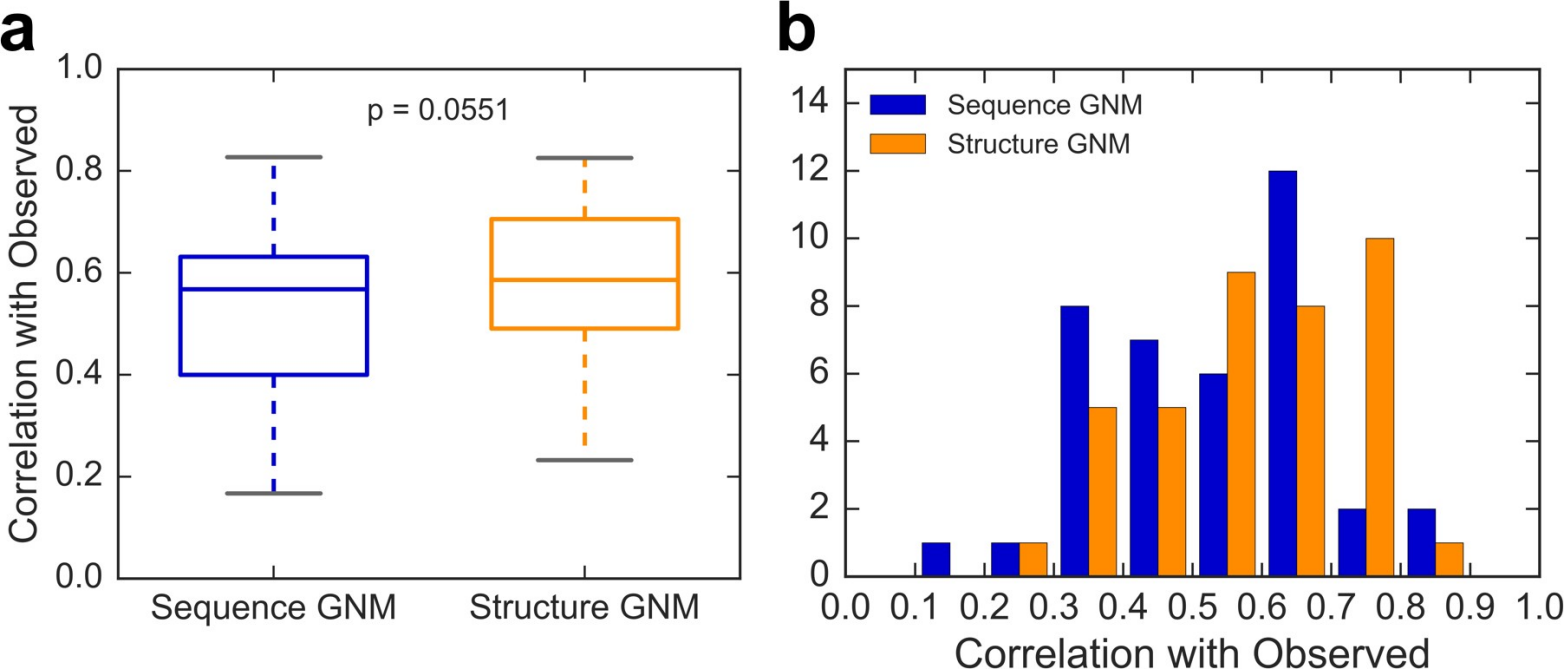
Comparison of B-factors obtained by GNM and Seq-GNM with experimental B-factors. (a) Boxplot showing the correlation of predicted B-factors by the Seq-GNM with experimentally observed B-factors (blue) in comparison to that of the GNM obtained from structure (orange) for a subset of 39 structures with resolution better than 2.0 Å. (b) A distribution plot of the same correlations binned into 10 bins with sizes of 0.1. A student t-test reveals no significant difference between the two distributions (p = 0.055) indicating that the Seq-GNM is producing competitive results compared to the original GNM from structure. The mean correlation of the Seq-GNM is 0.53 while that of the GNM from structure is 0.58.

As shown in Fig 3A, boxplot distributions reveal that correlations are not significantly different between the sequence and structure GNM (p = 0.055 in a student t-test). The structure GNM appears to perform only slightly better than the Seq-GNM. Fig 3B shows the same distribution separated into 10 individual bins of size 0.1. The overall shapes of the two distributions are similar, except for the exaggerated relative lower second peak of the Seq-GNM at 0.4. It should also be noted that for these cases where Seq-GNM had low correlations, the EC threshold could be tuned to yield much higher correlations. If this were done on a case-by-case basis, the overall correlation distributions would be even more similar. Thus, the EC threshold may be used as a tuning parameter to enhance the correlation coefficient for purposes of model optimization.

**Fig 4:**
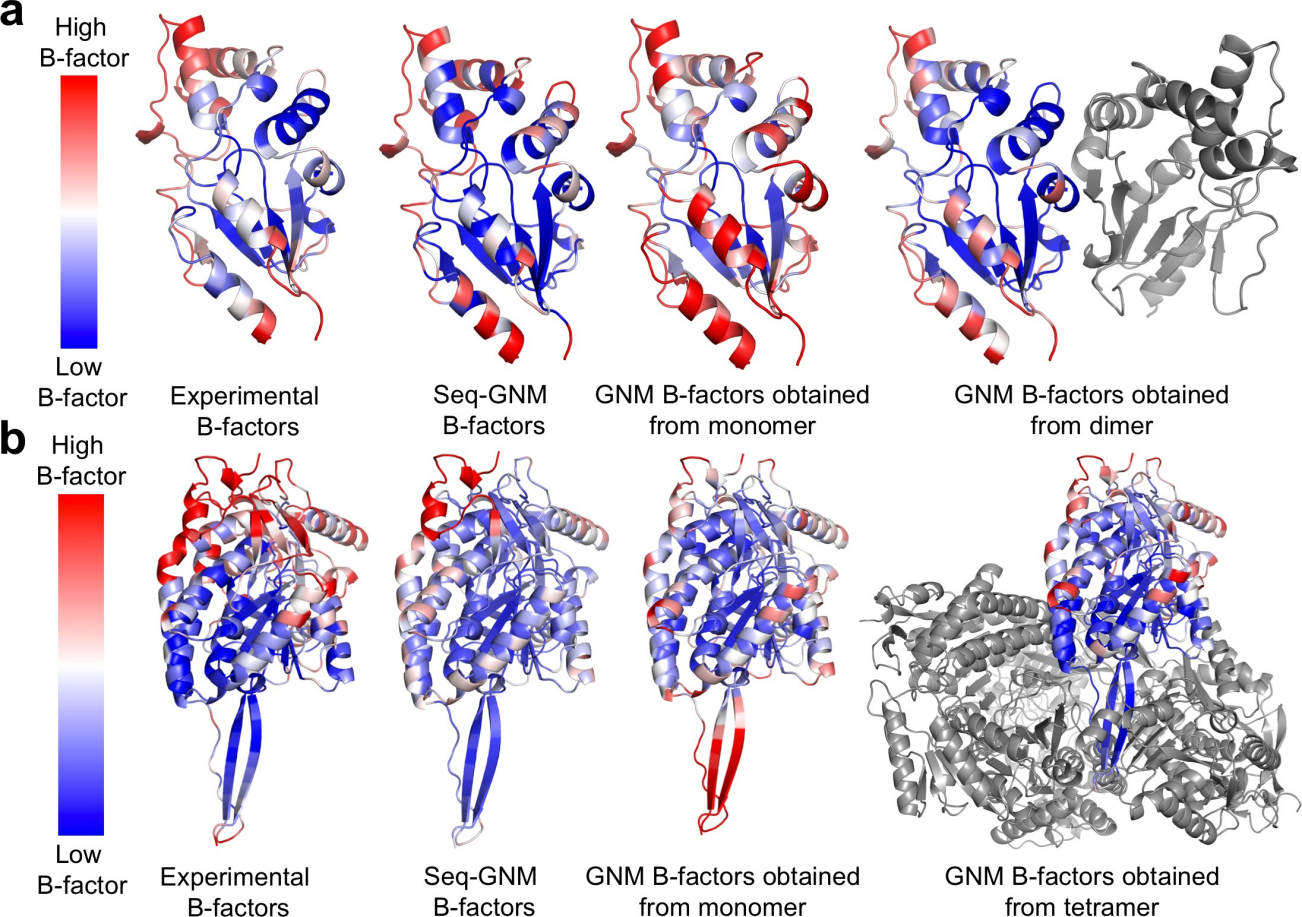
Comparison of B-factors obtained from experiments, Seq-GNM, GNM from monomeric structure, and GNM from oligomeric structure. B-factors are shown on the respective structures for (a) 5'(3')-deoxyribonucleotidase (2JAO) and (b) Aldehyde Dehydrogenase 7A1 (2J6L). (a) The correlation of Seq-GNM to experimental B-factors is 0.83 while correlation of GNM B-factors obtained from monomer to experimental B-factors is 0.63. When dimer a is used for GNM analysis the correlation of GNM B-factors obtained from monomer to experimental B-factors increased to 0.72. (b) The correlation of Seq-GNM to experimental B-factors is 0.61 while correlation of GNM B-factors obtained from monomer to experimental B-factors is 0.37. When a tetramer is used for GNM analysis the correlation of GNM B-factors obtained from monomer to experimental B-factors increase to 0.76. The change in correlation for GNM between monomer and oligomer clearly shows the drawback for dependence on the crystal structure of biounits. However, Seq-GNM captures the interface B-factors correctly.

Interestingly, for the cases where predicted B-factors by Seq-GNM yielded significantly better correlations with the experimental B-factors than those obtained by GNM from structures, we observed that biological units of these proteins are assigned as oligomeric forms. While predicted B-factors obtained using Seq-GNM does not retain this information, it successfully predicts the experimentally low B-factor values of interface positions as shown for protein 5'(3')-deoxyribonucleotidase (2JAO) and protein aldehyde Dehydrogenase 7A1 (2J6L) in Fig 4. It is indeed shown in earlier work of direct contact analysis that co-evolution can identify positions of protein interfaces and protein-protein interaction partners and successfully reconstruct protein complexes and interaction network [23,30,48]. Thus, it is not surprising to see that it yields good correlations with the experimental B-factors. Conversely, predicted B-factors from structure can only improve when the oligomeric structure is used for the GNM analysis.

**Fig 5:**
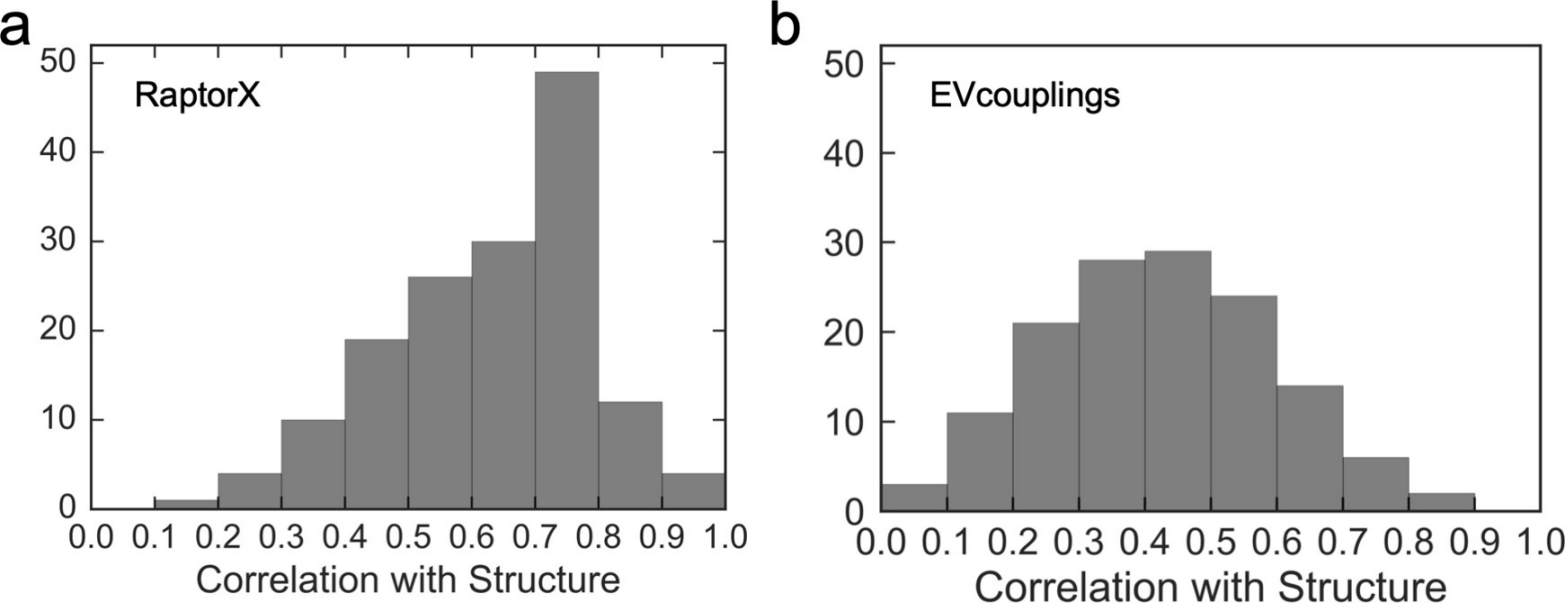
Distribution of correlation coefficients. The distribution of correlation coefficients between B-factors from Seq-GNM and GNM from structure. (a) The average correlation coefficient is 0.63 with RaptorX EC values. (b) The average correlation coefficient is 0.43 by using EVcouplings EC values.

Even when using high-resolution X-ray structures, there is still some uncertainty about the realistic nature of crystallographic B-factors. For this reason, we thought a more plausible way to determine the efficacy of the Seq-GNM was to compare it directly with the structure GNM. The structure GNM is a robust method to describe thermal fluctuations in a protein, and in many cases, it performs as good or better than the ANM or MD [47,49]. We systematically evaluated the performance of the Seq-GNM and structure GNM for the entire set of 139 structures and obtained the correlation coefficients for each protein (Fig 5).

The average correlation of B-factors between the Seq-GNM and structure GNM model is 0.63 when using EC contacts from RaptorX and 0.43 when using contacts from EVcouplings. As seen in Fig 5A, the distribution of correlation coefficients increases until 0.8, and then subsequently decreases. Interestingly, there are still an appreciable number of sequences yielding high correlations from 0.8 to 1.0. A distinguishing feature of the distribution is the pronounced peak in the bin from 0.7 to 0.8, indicating that significant fraction of our data set yields high correlations between 0.7 and 0.8. This is evidence that the Seq-GNM is efficiently capturing protein dynamics and supports the theory that ECs can be used as a substitute to 3D structure contacts in the GNM and still produce reliable dynamics profiles. The results of Seq-GNM based on contacts predicted by RaptorX usually yields B-factors that are closer to experimental B-factors as it uses structural information in its neural networks leading to better EC values and correlations with structure [44].

### Assessing nSNV phenotypes using the Seq-GNM

Crystallographic B-factors have previously been used to assess the impact of nSNVs on protein function [18,50–54]. A study [51] found that mutations on lysozyme that impaired function exhibited lower than average temperature factors, suggesting that rigid sites on the protein are more susceptible to destabilizing nSNVs than flexible sites [55]. Another study revealed a relationship between crystallographic B-factors and the impact of nSNVs on protein function [56]. A commonly used tool to diagnose neutral and disease associated nSNVs, PolyPhen-2, uses evolutionary information, structural information, and crystallographic B-factors in its prediction model [49]. These studies indicate that crystallographic B-factors can be used to predict the tolerance of a given residue to an nSNV (i.e., whether or not the occurrence of an nSNV would impact function).

We investigated whether B-factors predicted by the Seq-GNM were indicative of biological phenotype for nSNVs in the human population. A total of 738 nSNVs were mapped to the 139 enzymes, where 436 are disease-associated and 302 are neutral. S1 Table shows the number of disease and neutral nSNVs that occur on each protein. The Seq-GNM (using EC contacts from RaptorX and EVcouplings) was computed systematically for all 139 enzymes to obtain their dynamics profiles. The theoretical B-factors scores were converted into a percentile rank so that the values could be compared across different proteins.

We initially looked at two human enzymes, human lysozyme (PDB: 1C7P) and human cytochrome reductase (PDB: 1UMK). They were chosen because they were short proteins that each contain a disease and neutral nSNV. Human lysozyme is a glycoside hydrolase that functions in the immune system by causing damage to cell walls of bacteria. Human cytochrome b5 reductase is involved in many oxidation/reduction reactions including converting methemoglobin to hemoglobin [55].

**Fig 6:**
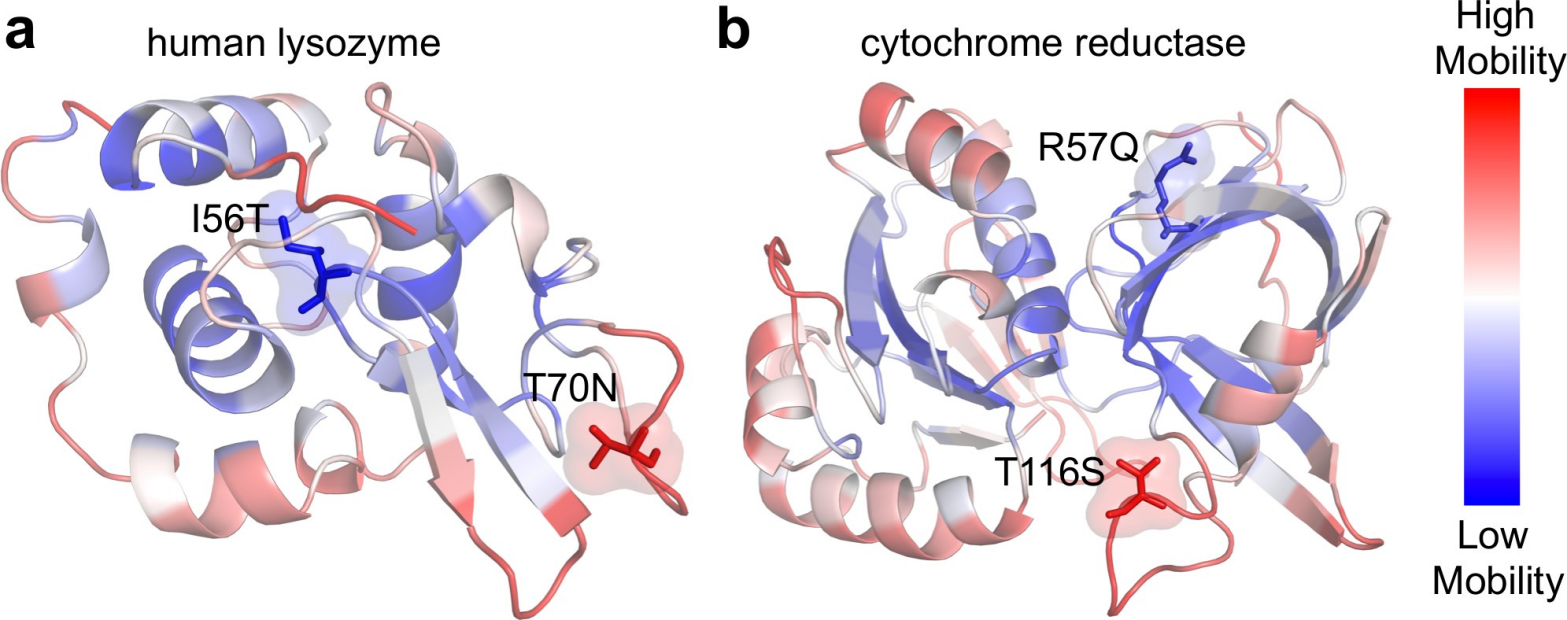
Comparison of theoretical B-factors on disease versus neutral mutant sites. A ribbon diagram for two human enzymes, human lysozyme (a) and cytochrome reductase (b) colored according to their predicted B-factors by the Seq-GNM. Red indicates high mobility sites, and blue indicates low mobility sites. Each protein contains two known nSNVs. I56T and R57Q are disease-associated, and they occur on low mobility (rigid) sites. Conversely, the neutral nSNVs T116S and T70N occur on high mobility sites.

Each structure is color-coded according to its theoretical B-factor profile on a spectrum of blue–white–red. Sites that exhibit high mobility (flexible) are red, and sites that have low mobility (rigid) are blue. Regions that are characterized by low mobility are usually important for maintaining stability and function, thus a mutation could act to destabilize the protein and impair its function. Fig 6A show the disease mutation I56T occurring on a rigid site with a B-factor of 0.0075. The neutral mutation T70N has a B-factor of 0.96 indicating that it is a highly mobile site. Both I56T and T70N occur on loop regions. Although loops are generally more flexible, three alpha-helical domains encompass the loop containing I56T, which implies that it may be involved in interactions that contribute to stabilizing the functional conformation. Thus, the I56T mutation may disrupt these critical interactions and impair the enzymatic function. In the case of cytochrome reductase (Fig 6B), the disease mutation R57Q is also on a rigid site with a B-factor of 0.14. Instead of being located near the core, R57Q is highly exposed protruding outwardly from a beta-barrel. However, since beta-barrels often harbor functional residues, the R57Q mutation may disrupt certain interactions critical for modulating function. The neutral mutation T116S is located on a loop and has a B-factor of 0.96, indicating that is it has a high mobility. In our earlier proteome wide study of over 100 human protein structures, we have shown that sites that are highly flexible (e.g., loop regions, or superficial sites) are typically more robust to mutations. Conversely, rigid sites are more susceptible to mutations that may disrupt function [18,19]. For these two cases, the B-factors produced by Seq-GNM successfully distinguished between the disease and neutral nSNVs, without using the 3D structures.

**Fig 7:**
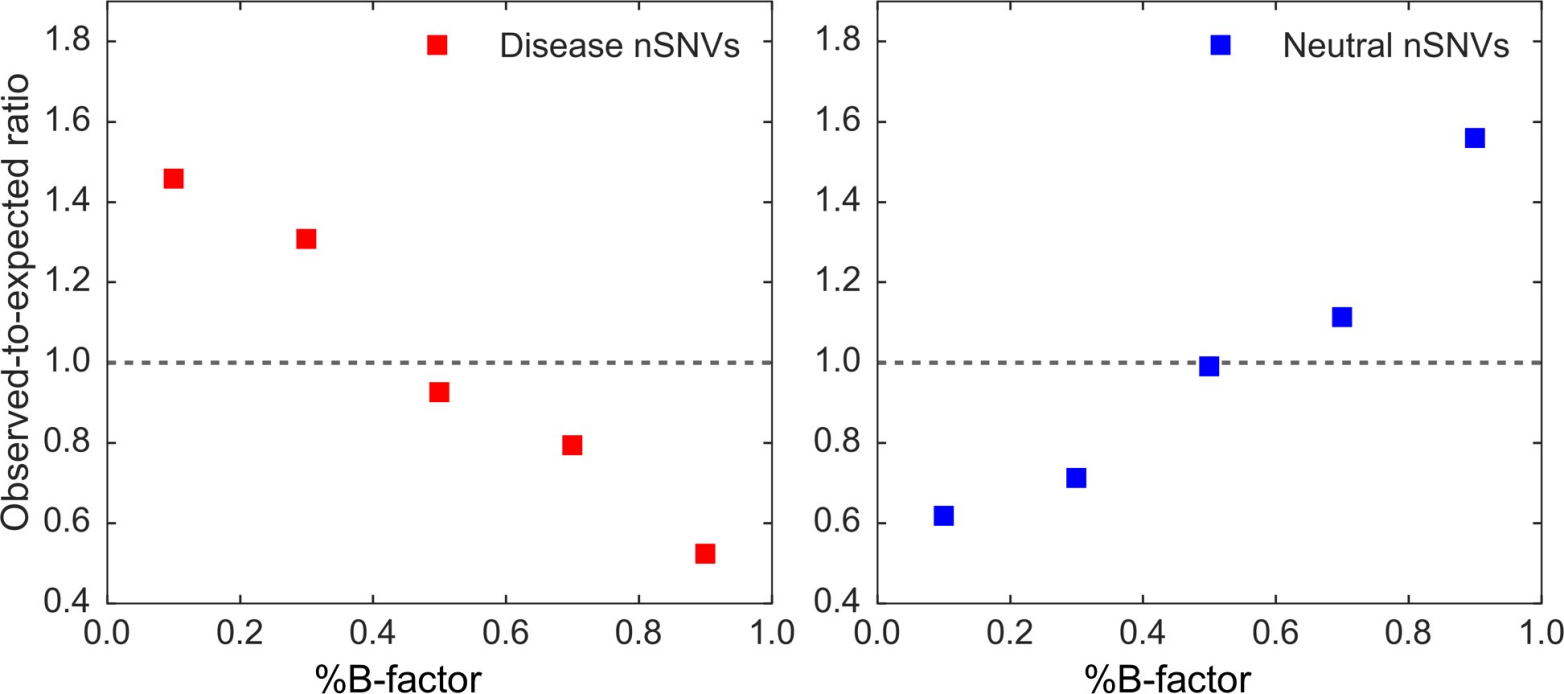
Observed to expected ratio plots for disease and neutral nSNVs. The relationship of observed-to-expected numbers between 436 disease nSNVs (red) and 302 neutral nSNVs (blue) from 139 human enzymes. The %B-factor scores derived from the Seq-GNM are binned into 5 bins of size 0.2.

These findings prompted us to analyze the proteome-wide set of 139 enzymes to determine if the B-factors were indicative of phenotype for all 436 disease and 302 neutral nSNVs. The raw B-factor values were converted into a percentile rank (%B-factor) and then binned into 5 bins of size 0.2. We computed the observed-to-expected ratio of B-factors, where the expected values were based on the B-factor distribution of all 51,618 sites across all 139 proteins, and the observed values were based on the B-factors of the 436 disease sites. The same process was done for the 302 neutral nSNVs. Under the null hypothesis that predicted B-factor of the disease associated nSNVs yields similar distribution of all the positions gathered from 139 enzyme sequences, the ratio of expected and observed sites harboring disease mutations for each %B-factor bin should be close to 1, which would imply that B-factor does not distinguish sites that are prone to disease. This is the null hypothesis that disease sites are distributed uniformly between sites with low and high mobility. However, the null hypothesis was rejected for the 436 disease nSNVs (p <0.001). Fig 7 shows the observed-to-expected ratio plot of disease and neutral nSNVs, which indicates that disease nSNVs are overabundant at low %B-factor sites (<0.4) and under abundant at high %B-factor sites. Conversely, neutral nSNVs are overabundant at high %B-factor sites (>0.6) and under abundant at low %B-factor sites. This evidence suggests that the occurrence of an nSNV on a site with a low B-factor is likely damaging based on the position irrelative of the substitution. This is in agreement with our previous proteome-wide study showing that substitutions at rigid sites are more often associated with diseases [18]. Conversely, an nSNV on a high B-factor site is usually benign. Low B-factors usually signify a residue that is crucial for modulating functional motions (e.g., a hinge). Thus, mutations on these sites can severely impact function. High B-factor sites are more flexible (e.g., loops) and more robust to mutations. Fig 7 suggest that it is possible to use the predicted B-factors to discriminate between disease and neutral nSNVs using co-evolution obtained from only multiple sequence alignment. Moreover, it can be used as an additional feature for *in silico* predictions [12].

**Fig 8:**
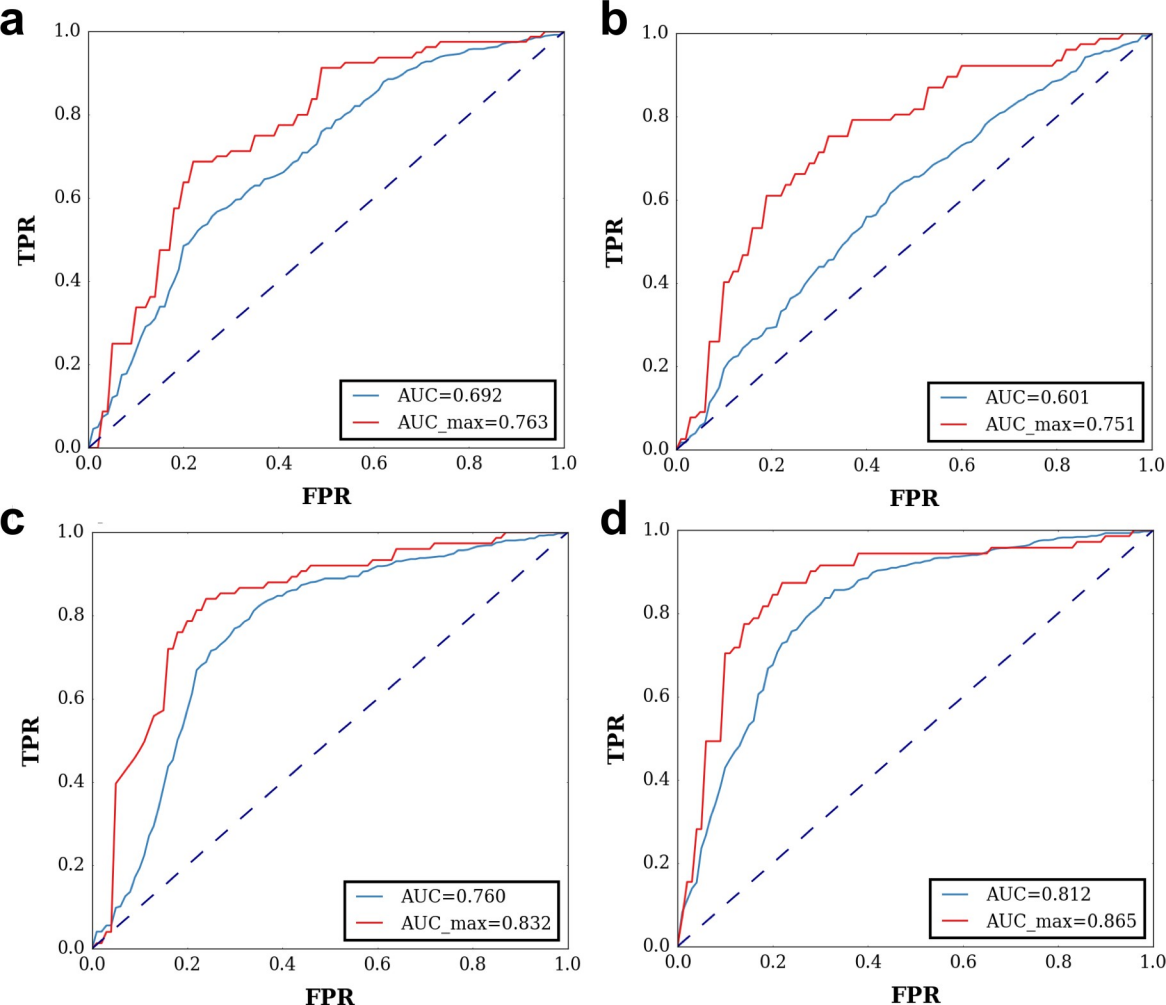
ROC curves for disease prediction performance comparing Seq-GNM, experimental B-factors and evolutionary parameters. ROC curves are plotted using 10 randomly selected training and testing data sets using 80%, and 20% of the data, respectively. (a) ROC curve of Seq-GNM. (b) ROC curve of experimental B-factors. (c) ROC curve of evolutionary parameters, where primate, mammal, and vertebrate fitch rates using Fitch Algorithm [57]; and Entropy2 are used as features for training. (d) ROC curve of evolutionary parameters used in (c) with the addition of Seq-GNM.

Predictive models were created using logistic regression as the classification algorithm, 80% of the data was used for training and 20% for testing for 10 randomized sets. Models were evaluated based on ROC curves and their respective area under curve (AUC), the best performance is labeled as AUC_max and average performance as AUC. Theoretical B-factors obtained by Seq-GNM, experimental B-factors, and evolutionary parameters were used as predictive variables for training and testing (Fig 8). Seq-GNM and experimental B-factors have similar performance (maximum AUC of best 0.76 and 0.75, respectively), with Seq-GNM overshadowing experimental B-factors on average (AUC of 0.69 and 0.60, respectively). The ~0.70 AUC of B-factors obtained from Seq-GNM is impressive, as it has been shown that majority of state-of-art methods also yields similar AUC in independent tests [5,13]. Moreover, incorporation of Seq-GNM as an additional feature with evolutionary parameters resulted in higher prediction performance. While the AUC scores obtained using the evolutionary features for classification gives 0.76, this is increased to 0.81 after including the B-factors of Seq-GNM (Fig 8C and 8D). This result also demonstrates the efficacy of Seq-GNM in disease prediction as a complementary metric to other metrics used as features in classifiers.

We also compared the performance of Seq-GNM with common *in silico* prediction tools like Polymorphism Phenotyping v2 (PolyPhen-2), and Sorting Intolerant from Tolerant (SIFT) [6,58]. The accuracy, sensitivity, and selectivity of disease predictions for nSNVs with experimental B-factors, B-factors from SIFT, PolyPhen-2, evolutionary parameters, and Seq-GNM are tabulated in Table 1. The accuracy of Seq-GNM using both EC values from EVcouplings and RaptorX is ~0.70. This accuracy is similar to using experimental B-factors for prediction (0.69) and also very close to prediction with evolutionary parameters (0.75), suggesting that Seq-GNM allows us to incorporate protein dynamics in nSNV predictions when the 3D experimental structures are not available. Moreover, accuracy of Seq-GNM approach is greater than SIFT (0.65) and PolyPhen-2 (0.64). Interestingly, Seq-GNM obtained by EVcouplings and RaptorX yields similar accuracies indicating that evolutionary couplings without the inclusion of structure could be utilized to predict B-factors to include as a feature to *in silico* prediction tools. Seq-GNM sensitivity (~0.90) surpasses other methods (0.80 for SIFT, 0.63 for PolyPhen-2, and 0.85 for evolutionary parameters), but it has a shortcoming in selectivity (~0.36) as other methods reach higher (~0.59). Conversely, training Seq-GNM combined with evolutionary parameters enhances the selectivity (0.66) to its highest value compared to others. Seq-GNM with evolutionary parameters predicted disease related nSNVs with accuracy 0.78 and sensitivity of 0.84, reaching beyond predictions of other metrics solely. These results suggest the incorporation of Seq-GNM with other prediction metrics can augment accuracy, sensitivity, and selectivity of prediction.

**Table 1:** The disease prediction data showing the accuracy, sensitivity, and the selectivity of Seq-GNM compared with experimental B-factors, SIFT, PolyPhen-2 and evolutionary parameters.

Classifier Feature and Methods	Accuracy	Sensitivity	Selectivity
Experimental B-factors	0.69	0.88	0.35
SIFT	0.65	0.70	0.56
PolyPhen-2	0.64	0.63	0.64
Seq-GNM B-factors (EVcouplings)	0.70	0.89	0.37
Seq-GNM B-factors (RaptorX)	0.71	0.91	0.35
Evolutionary parameters	0.75	0.85	0.58
Seq-GNM (EVcouplings) with evolutionary parameters	0.78	0.84	0.66

Prediction accuracy of Seq-GNM is further tested using 323 nSNVs (187 disease-associated, 136 neutral) of 22 proteins where their 3D experimental structures are not available (S2 Table). We used the trained classifier model of Seq-GNM B-factors for this test. While the B-factors obtained solely from Seq-GNM are used, it reached an accuracy, sensitivity, and selectivity of 0.82, 0.82, 0.83, respectively. This result further suggests that Seq-GNM allows us to incorporate protein dynamics as additional feature in *in silico* prediction tools without a known 3D structure.

## Discussion

While we and others [5,19,59–63] have shown that the integration of conformational dynamics into genomic analysis will help next generation of approaches to predict the impact of novel missense mutations on the human proteome, the inherent limitations in the availability of 3D structures compared to the vast number of sequences must be addressed. This begs the question: how can protein dynamics be used in genome-wide analysis to predict functional impacts of nSNVs? There is, therefore, a need to be able to obtain protein dynamics by leveraging only sequence information, without *a priori* knowledge of a 3D structure. For this reason, we have developed this novel method to estimate the dynamics profile of a protein by using only a sequence as input. The method uses the coevolution of amino acids through multiple sequence (which tend to be spatially close in the 3D tertiary structure) and a simple Gaussian network model (GNM) to obtain dynamics. The original GNM based on the 3D structure is well-known for its ability to describe residue dynamics profiles due to thermal motions in proteins (i.e., B-factors). We showed that our sequence-based GNM model is able to adequately reproduce the mean-square fluctuations (B-factors) calculated by the original GNM, particularly outperforms for the cases where biological functional state is oligomeric. Our estimates of B-factors for a proteome-wide set of proteins exhibited good correlation with the structure GNM. Moreover, our estimated B-factors were in reasonable agreement with crystallographic B-factors for many cases. To address the issue of how protein dynamics can determine the impact of nSNVs across the genome where there are no known 3D structures, we tested the ability of our predicted dynamics from the Seq-GNM to assess nSNV phenotypes. A plot of the observed-to-expected ratio of the predicted B-factors revealed distributions of disease and neutral nSNVs that are similar to those in a previous protein dynamics analysis work [18]. The predicted B-factors using the Seq-GNM was able to discriminate between disease and neutral nSNVs with an accuracy of 0.70 and incorporating the Seq-GNM predicted B-factors with evolutionary parameters increased overall accuracy to 0.78. This analysis demonstrates that the Seq-GNM makes it possible to obtain estimates of dynamics without using a 3D structure, which will allow for the integration of conformational dynamics into large-scale analysis of genomic variants.

## Methods

### Dataset

A curated set of 139 structures was selected for several reasons. First, they have high query coverage (>80%) and sequence identity (>80%) as found from a BLAST search, and the structures had already been modeled using the Modeller software package [64] to account for any missing residues. Second, genetic variants were previously mapped onto these structures, such that the positions containing known nSNVs were already determined, enabling us to easily compare our results using sequence coevolution with the genetic variation data. A total of 738 genetic variants were obtained from the HumVar database [58], which was comprised of 436 disease and 302 neutral nSNVs. Finally, the structures were either monomers or the single-chain unit of a multimer with <600 residues, allowing for tractable calculations of residue coevolution using the RaptorX web server [42,44], and EVfold (EVcouplings) [21]. A table summarizing the dataset is presented in S1 Table.

### Obtaining coevolved residues

The amino acid sequence from each of the 139 structures was used as input for the evolutionary coupling (EC) analysis. The choice of taking the amino acid sequence from the structure was done so that the predicted EC contacts could be compared directly to the experimentally observed structure contacts as verification that the model was producing realistic contact maps. Moreover, the theoretical B-factors predicted by our sequence-based model could be directly compared to the experimental B-factors for each protein. If the structure was unknown, however, sequence databases (e.g. UniProt, PFAM, etc.) could be used. The PDB sequences were given to the RaptorX web server [42,43], which computed the relative probability of each residue pair *i*, *j* of being in 3D contact based on their coevolution strength. The sequences were also used to generate MSAs using phmmer [65]. Using MSAs, DI values are calculated by EVcouplings. In order to ensure consistency between different proteins of varying lengths, we converted the raw scores into percentile ranks. We then used a threshold value, taking only the top scoring evolutionary couplings (i.e., the strongest couplings are more likely to be in spatial contact). An optimized threshold value was systematically evaluated and is discussed in the Methods.

### Sequence-based GNM model (Seq-GNM)

The Gaussian network model (GNM) is an isotropic approach based on the contact topology of a crystal protein structure to obtain the equilibrium fluctuations of residues due to thermal motion. It uses a specified cutoff distance to define interacting pairs that are connected by springs with a single-parameter harmonic potential. In this structure-based GNM, the interacting residue pairs within the cutoff range are represented as contacts in the Kirchhoff (connectivity matrix).

In the proposed sequence-based GNM (Seq-GNM) approach we will instead use coevolving residue pairs (evolutionary couplings) as contacts in the Kirchhoff. In this way, the 3D structure is no longer a prerequisite to form a GNM. To construct the Kirchhoff, a threshold is defined where any evolutionary coupling scores above that threshold are sufficiently coupled such that they are spatially close in 3D structure. If a given evolutionary coupling pair meets the threshold criteria, it is assigned a value in the Kirchhoff for non-bonded contacts of –1 multiplied by its evolutionary coupling score (i.e., –1×EC_score_). This will permit that the strength of each connection will attenuate proportionally to the evolutionary coupling strength. The Kirchhoff can be decomposed into the individual contributions from the bonded contacts representing the chain connectivity (Rouse chain) and that from the non-bonded contacts [56]. In the Seq-GNM the contribution of non-bonded contacts to the Kirchhoff is constructed according to

$$\Gamma_{ij}^{nb}=\left\{ \begin{array}{l} -1\times {EC}_{score},\;i\neq j\;evolutionary\;coupling\\ \;0,\;i\neq j\;no\;coupling\\ -\sum_{i,i\neq j}\Gamma_{ij},\;i=j \end{array}\right.$$ (1)

For the local chain connectivity (Rouse chain), we don’t take into account evolutionary couplings, and matrix was constructed such that every residue pair *i*, *i* ± 1 to *i*, *i* ± 3 is in contact as

$$\Gamma_{ij}^{cc}=\left\{ \begin{array}{l} \;-1,\;i\neq j\;and\;\sum_{i|k=1,2,3}^{L}i,i\pm k\\ \;0,\;i\neq j\;else\\ –\sum_{i,i\neq j}\Gamma_{ij},\;i=j \end{array}\right.$$ (2)

Then the overall Kirchhoff is the combination of the two contributions $\Gamma_{ij}=\Gamma_{ij}^{cc}+\Gamma_{ij}^{nb}$. The vibrational dynamics due to thermal fluctuations can then be evaluated in the same way as the original GNM by inverting the Kirchhoff matrix. The magnitude of mean-square fluctuations is then written in terms of the inverse Kirchhoff as

$$\langle {\left(\Delta R_{i}\right)}^{2}\rangle ≅{\left[\Gamma^{-1}\right]}_{ii}$$ (3)

**Fig 9:**
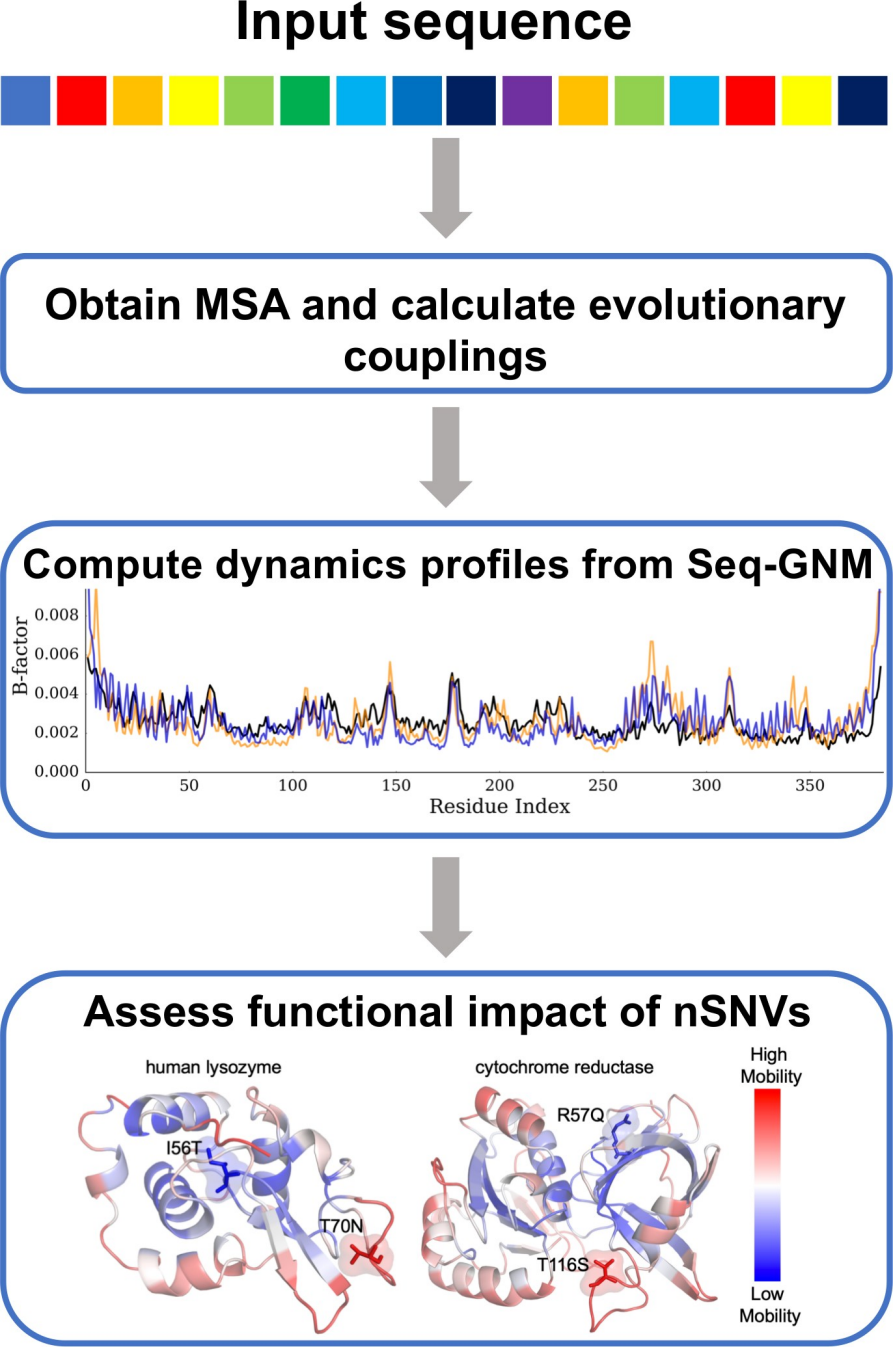
Flowchart of Seq-GNM method for nSNV predictions. A workflow of our method to use predicted evolutionary couplings to determine protein dynamics and assess the functional impact of nSNVs. The initial input is an amino acid sequence, which is used to obtain MSA. Using MSA evolutionary coupling pairs are predicted through RaptorX and EVcouplings. The high scored evolutionary coupling pairs are assigned as contacts in our Seq-GNM to compute the dynamics profile of each protein. The dynamic profiles obtained from Seq-GNM can give insight into the functional impact of nSNVs. This was done for a curated set of 139 structures.

This is proportional to the Debye-Waller temperature factors or B-factors, which describe the attenuation of X-ray scattering due to the thermal motions of atoms (*B_i_* = 8*π*^2^⟨(*Δ**R**_i_*)^2^⟩/3). Here there is no single-parameter force constant as in the GNM obtained from structure [52], and the pair-wise interactions are simply the strength of the evolutionary couplings as given by their ranked scores. The theoretical predictions of our Seq-GNM can be compared to the predictions of the original GNM obtained from structure as well as observed crystallographic B-factors. A general workflow of our method is presented as a flow diagram in Fig 9.

### Optimizing threshold value for EC scores

**Fig 10:**
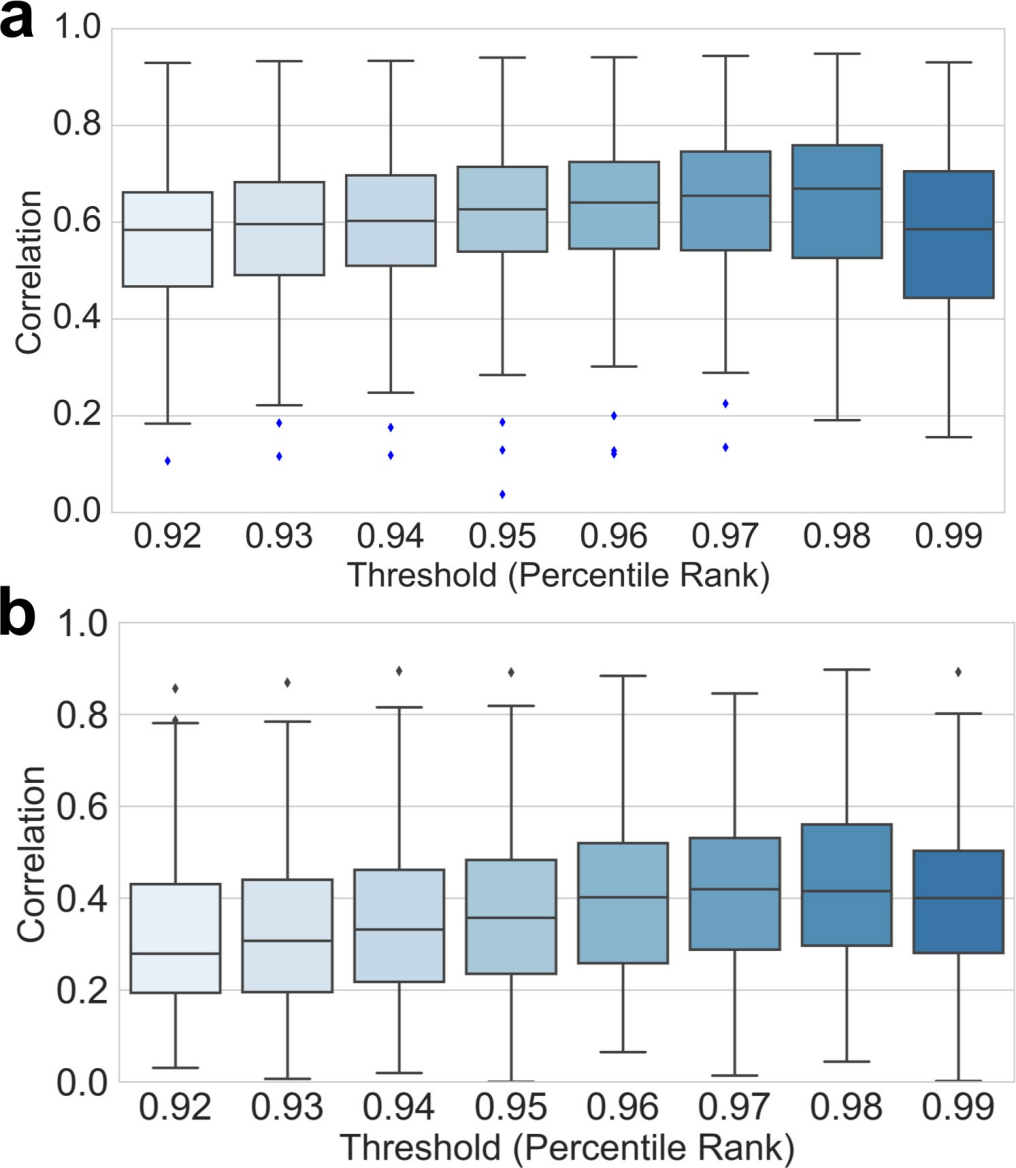
Comparison of theoretical B-factors. Boxplots comparing the correlations of predicted B-factors by our Seq-GNM for (a) RaptorX and (b) EVcouplings with that of the structural GNM for all 139 structures using a constant threshold for EC contacts. The GNM analysis is conducted 8 times using a constant threshold (between 0.92 and 0.99) each time. The best average correlations are produced when the constant threshold value of 0.98 is used.

To ensure consistency when analyzing different proteins with varying lengths, we converted the raw scores of evolutionary couplings (EC) into a percentile rank. We computed the Seq-GNM for all 139 structures using a constant threshold percentile rank EC value to assign contacts and measured the correlation between the B-factors predicted by our Seq-GNM to the GNM obtained from structure. We used only the top percentile EC scores predicted by RaptorX and EVcouplings as predicted contacts, because only certain fraction of high EC scores are true native contacts in 3D structure, largely due to noisy artifacts in the MSA such as the transitivity of correlations and phylogeny. To determine the optimal threshold value, we tested a range of threshold values from 0.92 to 0.99. A threshold value ≤0.92 yields superfluous contacts leading to a noisy contact map, and thus, a lower overall correlation (Fig 10). Conversely, a threshold value ≥0.99 gives a deficient number of contacts, which yields an excessively sparse contact map and a lower overall correlation. As Fig 10 shows, a threshold value of 0.98 produced the best overall correlation with the GNM from structure and, thus, was taken to be the optimal threshold value used in the analysis.

## Supporting information

S1 Table List of 139 proteins used in this study. Proteins used to compute theoretical B-factors based on Seq-GNM and GNM obtained from structure. B-factor correlation values between GNM obtained from structure to experimental (Str GNM-Expt), Seq-GNM to experimental (Seq GNM-Expt), and Seq-GNM to GNM obtained from structure (Seq GNM-Str GNM) are given for two methods (RaptorX, EVfold (EVcouplings)).
(XLSX)

S2 Table List of proteins without a known structure with disease related nSNVs used for prediction. Seq-GNM predictions by using a set of 323 nSNVs without a known 3D structure from 22 proteins. The protein identifiers, residue numbers, amino acid substitutions Seq-GNM scores, Seq-GNM predictions, and ground truth are provided.
(XLSX)

## References

[1] Berman HM, Westbrook J, Feng Z, Gilliland G, Bhat TN, Weissig H, et al The Protein Data Bank. Nucleic Acids Res. 2000;28: 235–242. 10592235 10.1093/nar/28.1.235 PMC102472

[2] Li MJ, Wang P, Liu X, Lim EL, Wang Z, Yeager M, et al GWASdb: a database for human genetic variants identified by genome-wide association studies. Nucleic Acids Res. 2012;40: D1047–D1054. 10.1093/nar/gkr1182 22139925 10.1093/nar/gkr1182 PMC3245026

[3] Xie L, Ge X, Tan H, Xie L, Zhang Y, Hart T, et al Towards Structural Systems Pharmacology to Study Complex Diseases and Personalized Medicine. PLOS Computational Biology. 2014;10: e1003554 10.1371/journal.pcbi.1003554 24830652 10.1371/journal.pcbi.1003554 PMC4022462

[4] Kumar S, Sanderford M, Gray VE, Ye J, Liu L. Evolutionary Diagnosis Method for Variants in Personal Exomes. Nat Methods. 2012;9 10.1038/nmeth.2147 22936163 10.1038/nmeth.2147 PMC3810944

[5] Kumar A, Butler BM, Kumar S, Ozkan SB. Integration of structural dynamics and molecular evolution via protein interaction networks: a new era in genomic medicine. Current opinion in structural biology. 2015;35: 135–142. 10.1016/j.sbi.2015.11.002 26684487 10.1016/j.sbi.2015.11.002 PMC4856467

[6] Ng PC, Henikoff S. SIFT: predicting amino acid changes that affect protein function. Nucleic Acids Res. 2003;31: 3812–3814. 12824425 10.1093/nar/gkg509 PMC168916

[7] Pers TH, Timshel P, Hirschhorn JN. SNPsnap: a Web-based tool for identification and annotation of matched SNPs. Bioinformatics. 2015;31: 418–420. 10.1093/bioinformatics/btu655 25316677 10.1093/bioinformatics/btu655 PMC4308663

[8] Choi Y, Sims GE, Murphy S, Miller JR, Chan AP. Predicting the Functional Effect of Amino Acid Substitutions and Indels. PLOS ONE. 2012;7: e46688 10.1371/journal.pone.0046688 23056405 10.1371/journal.pone.0046688 PMC3466303

[9] Reva B, Antipin Y, Sander C. Predicting the functional impact of protein mutations: application to cancer genomics. Nucleic Acids Res. 2011;39: e118–e118. 10.1093/nar/gkr407 21727090 10.1093/nar/gkr407 PMC3177186

[10] Wilkins A, Erdin S, Lua R, Lichtarge O. Evolutionary Trace for Prediction and Redesign of Protein Functional Sites. Methods Mol Biol. 2012;819: 29–42. 10.1007/978-1-61779-465-0_3 22183528 10.1007/978-1-61779-465-0_3 PMC4892863

[11] González-Pérez A, López-Bigas N. Improving the Assessment of the Outcome of Nonsynonymous SNVs with a Consensus Deleteriousness Score, Condel. Am J Hum Genet. 2011;88: 440–449. 10.1016/j.ajhg.2011.03.004 21457909 10.1016/j.ajhg.2011.03.004 PMC3071923

[12] Kumar S, Dudley JT, Filipski A, Liu L. Phylomedicine: an evolutionary telescope to explore and diagnose the universe of disease mutations. Trends in Genetics. 2011;27: 377–386. 10.1016/j.tig.2011.06.004 21764165 10.1016/j.tig.2011.06.004 PMC3272884

[13] Katsonis P, Koire A, Wilson SJ, Hsu T-K, Lua RC, Wilkins AD, et al Single nucleotide variations: Biological impact and theoretical interpretation. Protein Science. 2014;23: 1650–1666. 10.1002/pro.2552 25234433 10.1002/pro.2552 PMC4253807

[14] Mahmood K, Jung C, Philip G, Georgeson P, Chung J, Pope BJ, et al Variant effect prediction tools assessed using independent, functional assay-based datasets: implications for discovery and diagnostics. Hum Genomics. 2017;11 10.1186/s40246-017-0104-8 28511696 10.1186/s40246-017-0104-8 PMC5433009

[15] Gray VE, Kukurba KR, Kumar S. Performance of computational tools in evaluating the functional impact of laboratory-induced amino acid mutations. Bioinformatics. 2012;28: 2093–2096. 10.1093/bioinformatics/bts336 22685075 10.1093/bioinformatics/bts336 PMC3413386

[16] Miller M, Bromberg Y, Swint-Kruse L. Computational predictors fail to identify amino acid substitution effects at rheostat positions. Scientific Reports. 2017;7: 41329 10.1038/srep41329 28134345 10.1038/srep41329 PMC5278360

[17] Kumar A, Glembo TJ, Ozkan SB. The Role of Conformational Dynamics and Allostery in the Disease Development of Human Ferritin. Biophysical Journal. 2015;109: 1273–1281. 10.1016/j.bpj.2015.06.060 26255589 10.1016/j.bpj.2015.06.060 PMC4576160

[18] Nevin Gerek Z, Kumar S, Banu Ozkan S. Structural dynamics flexibility informs function and evolution at a proteome scale. Evolutionary applications. 2013;6: 423–433. 10.1111/eva.12052 23745135 10.1111/eva.12052 PMC3673471

[19] Butler BM, Gerek ZN, Kumar S, Ozkan SB. Conformational dynamics of nonsynonymous variants at protein interfaces reveals disease association. Proteins: Structure, Function, and Bioinformatics. 2015;83: 428–435. 10.1002/prot.24748 PMC4505818 25546381

[20] Ponzoni L, Bahar I. Structural dynamics is a determinant of the functional significance of missense variants. PNAS. 2018; 201715896. 10.1073/pnas.1715896115 29610305 10.1073/pnas.1715896115 PMC5910821

[21] Marks DS, Colwell LJ, Sheridan R, Hopf TA, Pagnani A, Zecchina R, et al Protein 3D Structure Computed from Evolutionary Sequence Variation. PLOS ONE. 2011;6: e28766 10.1371/journal.pone.0028766 22163331 10.1371/journal.pone.0028766 PMC3233603

[22] Hopf TA, Schärfe CPI, Rodrigues JPGLM, Green AG, Kohlbacher O, Sander C, et al Sequence co-evolution gives 3D contacts and structures of protein complexes. eLife. 3 10.7554/eLife.03430 25255213 10.7554/eLife.03430 PMC4360534

[23] Morcos F, Pagnani A, Lunt B, Bertolino A, Marks DS, Sander C, et al Direct-coupling analysis of residue coevolution captures native contacts across many protein families. PNAS. 2011;108: E1293–E1301. 10.1073/pnas.1111471108 22106262 10.1073/pnas.1111471108 PMC3241805

[24] Marks DS, Hopf TA, Sander C. Protein structure prediction from sequence variation. Nature Biotechnology. 2012;30: 1072–1080. 10.1038/nbt.2419 23138306 10.1038/nbt.2419 PMC4319528

[25] Hopf TA, Colwell LJ, Sheridan R, Rost B, Sander C, Marks DS. Three-Dimensional Structures of Membrane Proteins from Genomic Sequencing. Cell. 2012;149: 1607–1621. 10.1016/j.cell.2012.04.012 22579045 10.1016/j.cell.2012.04.012 PMC3641781

[26] Taylor WR, Sadowski MI. Structural Constraints on the Covariance Matrix Derived from Multiple Aligned Protein Sequences. PLOS ONE. 2011;6: e28265 10.1371/journal.pone.0028265 22194819 10.1371/journal.pone.0028265 PMC3237328

[27] Sułkowska JI, Morcos F, Weigt M, Hwa T, Onuchic JN. Genomics-aided structure prediction. PNAS. 2012;109: 10340–10345. 10.1073/pnas.1207864109 22691493 10.1073/pnas.1207864109 PMC3387073

[28] Kim DE, DiMaio F, Wang RY-R, Song Y, Baker D. One contact for every twelve residues allows robust and accurate topology-level protein structure modeling. Proteins: Structure, Function, and Bioinformatics. 2014;82: 208–218. 10.1002/prot.24374 23900763 10.1002/prot.24374 PMC4128384

[29] Smock RG, Rivoire O, Russ WP, Swain JF, Leibler S, Ranganathan R, et al An interdomain sector mediating allostery in Hsp70 molecular chaperones. Molecular Systems Biology. 2010;6: 414 10.1038/msb.2010.65 20865007 10.1038/msb.2010.65 PMC2964120

[30] Juan D de, Pazos F, Valencia A. Emerging methods in protein co-evolution. Nature Reviews Genetics. 2013;14: 249–261. 10.1038/nrg3414 23458856 10.1038/nrg3414

[31] Schug A, Weigt M, Onuchic JN, Hwa T, Szurmant H. High-resolution protein complexes from integrating genomic information with molecular simulation. PNAS. 2009;106: 22124–22129. 10.1073/pnas.0912100106 20018738 10.1073/pnas.0912100106 PMC2799721

[32] Jana B, Morcos F, Onuchic JN. From structure to function: the convergence of structure based models and co-evolutionary information. Phys Chem Chem Phys. 2014;16: 6496–6507. 10.1039/c3cp55275f 24603809 10.1039/c3cp55275f

[33] Morcos F, Jana B, Hwa T, Onuchic JN. Coevolutionary signals across protein lineages help capture multiple protein conformations. PNAS. 2013;110: 20533–20538. 10.1073/pnas.1315625110 24297889 10.1073/pnas.1315625110 PMC3870752

[34] Sutto L, Marsili S, Valencia A, Gervasio FL. From residue coevolution to protein conformational ensembles and functional dynamics. PNAS. 2015;112: 13567–13572. 10.1073/pnas.1508584112 26487681 10.1073/pnas.1508584112 PMC4640757

[35] Kass I, Horovitz A. Mapping pathways of allosteric communication in GroEL by analysis of correlated mutations. Proteins. 2002;48: 611–617. 10.1002/prot.10180 12211028 10.1002/prot.10180

[36] Marks DS, Colwell LJ, Sheridan R, Hopf TA, Pagnani A, Zecchina R, et al Protein 3D Structure Computed from Evolutionary Sequence Variation. PLOS ONE. 2011;6: e28766 10.1371/journal.pone.0028766 22163331 10.1371/journal.pone.0028766 PMC3233603

[37] Morcos F, Pagnani A, Lunt B, Bertolino A, Marks DS, Sander C, et al Direct-coupling analysis of residue coevolution captures native contacts across many protein families. PNAS. 2011;108: E1293–E1301. 10.1073/pnas.1111471108 22106262 10.1073/pnas.1111471108 PMC3241805

[38] Jones DT, Buchan DWA, Cozzetto D, Pontil M. PSICOV: precise structural contact prediction using sparse inverse covariance estimation on large multiple sequence alignments. Bioinformatics. 2012;28: 184–190. 10.1093/bioinformatics/btr638 22101153 10.1093/bioinformatics/btr638

[39] Burger L, Nimwegen E van. Disentangling Direct from Indirect Co-Evolution of Residues in Protein Alignments. PLOS Computational Biology. 2010;6: e1000633 10.1371/journal.pcbi.1000633 20052271 10.1371/journal.pcbi.1000633 PMC2793430

[40] Park H, Ovchinnikov S, Kim DE, DiMaio F, Baker D. Protein homology model refinement by large-scale energy optimization. PNAS. 2018; 201719115. 10.1073/pnas.1719115115 29507254 10.1073/pnas.1719115115 PMC5866580

[41] Dunn SD, Wahl LM, Gloor GB. Mutual information without the influence of phylogeny or entropy dramatically improves residue contact prediction. Bioinformatics. 2008;24: 333–340. 10.1093/bioinformatics/btm604 18057019 10.1093/bioinformatics/btm604

[42] Ma J, Wang S, Wang Z, Xu J. Protein contact prediction by integrating joint evolutionary coupling analysis and supervised learning. Bioinformatics. 2015;31: 3506–3513. 10.1093/bioinformatics/btv472 26275894 10.1093/bioinformatics/btv472 PMC4838177

[43] Wang S, Li W, Zhang R, Liu S, Xu J. CoinFold: a web server for protein contact prediction and contact-assisted protein folding. Nucleic Acids Res. 2016;44: W361–W366. 10.1093/nar/gkw307 27112569 10.1093/nar/gkw307 PMC4987891

[44] Wang S, Sun S, Li Z, Zhang R, Xu J. Accurate De Novo Prediction of Protein Contact Map by Ultra-Deep Learning Model. PLOS Computational Biology. 2017;13: e1005324 10.1371/journal.pcbi.1005324 28056090 10.1371/journal.pcbi.1005324 PMC5249242

[45] Morcos F, Schafer NP, Cheng RR, Onuchic JN, Wolynes PG. Coevolutionary information, protein folding landscapes, and the thermodynamics of natural selection. PNAS. 2014;111: 12408–12413. 10.1073/pnas.1413575111 25114242 10.1073/pnas.1413575111 PMC4151759

[46] Carugo O. How large B-factors can be in protein crystal structures. BMC Bioinformatics. 2018;19 10.1186/s12859-018-2083-8 29471780 10.1186/s12859-018-2083-8 PMC5824579

[47] Kundu S, Melton JS, Sorensen DC, Phillips GN. Dynamics of Proteins in Crystals: Comparison of Experiment with Simple Models. Biophysical Journal. 2002;83: 723–732. 10.1016/S0006-3495(02)75203-X 12124259 10.1016/S0006-3495(02)75203-X PMC1302181

[48] Weigt M, White RA, Szurmant H, Hoch JA, Hwa T. Identification of direct residue contacts in protein–protein interaction by message passing. PNAS. 2009;106: 67–72. 10.1073/pnas.0805923106 19116270 10.1073/pnas.0805923106 PMC2629192

[49] Doruker P, Atilgan AR, Bahar I. Dynamics of proteins predicted by molecular dynamics simulations and analytical approaches: Application to α-amylase inhibitor. Proteins: Structure, Function, and Bioinformatics. 2000;40: 512–524. 10.1002/1097-0134(20000815)40:3<512::AID-PROT180>3.0.CO;2-M 10861943

[50] Chasman D, Adams RM. Predicting the functional consequences of non-synonymous single nucleotide polymorphisms: structure-based assessment of amino acid variation11Edited by F. Cohen. Journal of Molecular Biology. 2001;307: 683–706. 10.1006/jmbi.2001.4510 11254390 10.1006/jmbi.2001.4510

[51] Alber T, Sun DP, Nye JA, Muchmore DC, Matthews BW. Temperature-sensitive mutations of bacteriophage T4 lysozyme occur at sites with low mobility and low solvent accessibility in the folded protein. Biochemistry. 1987;26: 3754–3758. 10.1021/bi00387a002 3651410 10.1021/bi00387a002

[52] Elahian F, Sepehrizadeh Z, Moghimi B, Mirzaei SA. Human cytochrome b5 reductase: structure, function, and potential applications. Critical Reviews in Biotechnology. 2014;34: 134–143. 10.3109/07388551.2012.732031 23113554 10.3109/07388551.2012.732031

[53] Griffin JWD, Bradshaw PC. In silico prediction of novel residues involved in amyloid primary nucleation of human I56T and D67H lysozyme. BMC Structural Biology. 2018;18: 9 10.1186/s12900-018-0088-1 30029603 10.1186/s12900-018-0088-1 PMC6053722

[54] Gerek ZN, Ozkan SB. Change in Allosteric Network Affects Binding Affinities of PDZ Domains: Analysis through Perturbation Response Scanning. PLOS Computational Biology. 2011;7: e1002154 10.1371/journal.pcbi.1002154 21998559 10.1371/journal.pcbi.1002154 PMC3188487

[55] Tirion MM. Large Amplitude Elastic Motions in Proteins from a Single-Parameter, Atomic Analysis. Phys Rev Lett. 1996;77: 1905–1908. 10.1103/PhysRevLett.77.1905 10063201 10.1103/PhysRevLett.77.1905

[56] Bahar I, Atilgan AR, Erman B. Direct evaluation of thermal fluctuations in proteins using a single-parameter harmonic potential. Folding and Design. 1997;2: 173–181. 10.1016/S1359-0278(97)00024-2 9218955 10.1016/S1359-0278(97)00024-2

[57] Fitch WM. Toward Defining the Course of Evolution: Minimum Change for a Specific Tree Topology. Systematic Zoology. 1971;20: 406–416. 10.2307/2412116

[58] Adzhubei IA, Schmidt S, Peshkin L, Ramensky VE, Gerasimova A, Bork P, et al A method and server for predicting damaging missense mutations. Nature Methods. 2010;7: 248–249. 10.1038/nmeth0410-248 20354512 10.1038/nmeth0410-248 PMC2855889

[59] Nussinov R, Tsai C-J. Allostery in disease and in drug discovery. Cell. 2013;153: 293–305. 10.1016/j.cell.2013.03.034 23582321 10.1016/j.cell.2013.03.034

[60] Tsai C-J, Del Sol A, Nussinov R. Allostery: absence of a change in shape does not imply that allostery is not at play. Journal of molecular biology. 2008;378: 1–11. 10.1016/j.jmb.2008.02.034 18353365 10.1016/j.jmb.2008.02.034 PMC2684958

[61] Tsai C-J, Del Sol A, Nussinov R. Protein allostery, signal transmission and dynamics: a classification scheme of allosteric mechanisms. Molecular Biosystems. 2009;5: 207–216. 10.1039/b819720b 19225609 10.1039/b819720b PMC2898650

[62] Swint-Kruse L, Matthews KS, Smith PE, Pettitt BM. Comparison of simulated and experimentally determined dynamics for a variant of the LacI DNA-binding domain, NLac-P. Biophysical journal. 1998;74: 413–421. 9449341 10.1016/s0006-3495(98)77798-7 PMC1299393

[63] Tungtur S, Skinner H, Zhan H, Swint-Kruse L, Beckett D. In vivo tests of thermodynamic models of transcription repressor function. Biophysical chemistry. 2011;159: 142–151. 10.1016/j.bpc.2011.06.005 21715082 10.1016/j.bpc.2011.06.005 PMC3166966

[64] Narayanan Eswar, Ben Webb, Marti‐Renom Marc A., Madhusudhan M.S., Eramian David, Shen Min‐yi, et al Comparative Protein Structure Modeling Using Modeller. Current Protocols in Bioinformatics. 2014;15: 5.6.1–5.6.30. 10.1002/0471250953.bi0506s15 18428767 10.1002/0471250953.bi0506s15 PMC4186674

[65] Finn RD, Clements J, Eddy SR. HMMER web server: interactive sequence similarity searching. Nucleic Acids Res. 2011;39: W29–W37. 10.1093/nar/gkr367 21593126 10.1093/nar/gkr367 PMC3125773

